# Heterologous Prime-Boost Vaccination with MF59-Adjuvanted H5 Vaccines Promotes Antibody Affinity Maturation towards the Hemagglutinin HA1 Domain and Broad H5N1 Cross-Clade Neutralization

**DOI:** 10.1371/journal.pone.0095496

**Published:** 2014-04-22

**Authors:** Surender Khurana, Elizabeth M. Coyle, Milena Dimitrova, Flora Castellino, Karl Nicholson, Giuseppe Del Giudice, Hana Golding

**Affiliations:** 1 Division of Viral products, Center for Biologics Evaluation and Research (CBER), Food and Drug Administration (FDA), Bethesda, Maryland, United States of America; 2 Novartis Vaccines and Diagnostics Srl, Sienna, Italy; 3 Department of Infection, Inflammation, and Immunity, Maurice Shock Medical Sciences Building, University of Leicester, Leicester, United Kingdom; University of Cape Town, South Africa

## Abstract

In an open label clinical study (2007), MF59-adjuvanted hemagglutinin (HA) vaccine from H5N1-A/Vietnam/1194/2004 (clade 1) was administered to subjects previously vaccinated (primed) with clade 0 H5N3 (A/duck/Singapore/97) vaccine at least 6 years earlier (in 1999 or 2001). The primed individuals responded rapidly and generated high neutralizing antibody titers against the H5N1-Vietnam strain within 7 days of a single booster vaccination. Furthermore, significant cross-neutralization titers were measured against H5N1 clade 0, 1, and 2 viruses. In the current study, the impact of MF59 adjuvant during heterologous priming on the quality of humoral polyclonal immune response in different vaccine arms were further evaluated using real time kinetics assay by surface plasmon resonance (SPR). Total anti-H5N1 HA1 polyclonal sera antibody binding from the heterologous prime-boost groups after a single MF59-H5N1 boost was significantly higher compared with sera from unprimed individuals that received two MF59-H5N1 vaccinations. The antigen-antibody complex dissociation rates (surrogate for antibody affinity) of the polyclonal sera against HA1 of H5N1-A/Vietnam/1194/2004 from the MF59-H5N3 primed groups were significantly higher compared to sera from unadjuvanted primed groups or unprimed individuals that received two MF59-H5N1 vaccines. Furthermore, strong inverse correlations were observed between the antibody dissociation off-rates of the immune sera against HA1 (but not HA2) and the virus neutralization titers against H5 vaccine strains and heterologous H5N1 strains. These findings supports the use of oil-in-water-adjuvanted pandemic influenza vaccines to elicit long term memory B cells with high affinity BCR capable of responding to potential variant pandemic viruses likely to emerge and adapt to human transmissions.

## Introduction

Pandemic influenza preparedness is largely dependent on the immune status of the human population. In the case of seasonal influenza strains, pre-existing immunity is an important factor in reducing disease severity in most individuals. In the case of avian influenza (H5N1, H7N9, H9N2), there is little or no pre-existing antibody immunity in the human populations, which when combined with more pathogenic avian influenza virus (AIV) strains can lead to pandemic with high mortality rates. A vaccination strategy that could elicit long term immunity with a probability of some cross protection against emerging strains will be of great value and impact on global public health.

The concept of heterologous prime-boost protocols have been evaluated with vaccines against H5N1 avian influenza virus. In one such study, 54 individuals were vaccinated in 2007 with 7.5 µg of MF59-adjuvanted surface antigen H5N1 A/Vietnam/1194/2004 (clade 1), of whom 24 were primed earlier (1999 or 2001) with heterologous H5N3 vaccine (A/duck/Singapore/1997) either with MF59 adjuvant (12 subjects) or without any adjuvant (12 subjects). In previously primed individuals neutralization titers rose rapidly after a single H5N1-MF59 boost against homologous and heterologous (clade 0, 1, and 2) viruses. After 6 months, high titers of cross-reactive antibodies remained detectable among the MF59-adjuvanted H5N3 primed subjects [1], [2]. It was postulated that the remote vaccination with heterologous H5N3 subunit vaccine (with the MF59 adjuvant) resulted in expansion of long lived memory B cells that undergone maturation in germinal centers (GC) and could be quickly recalled after a boost with a different H5 strain.

We have previously studied the impact of oil-in-water adjuvant on the antibody epitope repertoire and polyclonal sera antibody affinity of anti-H5N1 and H1N1pdm009 humoral immune responses using whole genome phage display libraries (GFPDL), and Surface Plasmon Resonance (SPR) combined with recombinant hemagglutinin globular head domain (HA1) and stalk domain (HA2) proteins expressed in bacterial system[3] [4]. In the current study we explored the quality of the polyclonal sera in the heterologous prime-boost vaccine groups and the impact of MF59 adjuvant, using SPR real time kinetics assays to quantitate total antibody binding and polyclonal sera antibody affinity against recombinant HA1 and HA2 domains derived from the boosting H5N1 vaccine strain (A/Vietnam/1194/2004). Technically, since antibodies are bivalent, the proper term for their binding to multivalent antigens like viruses is avidity, but here we use the term affinity throughout since we do not describe any monovalent interactions. A strong correlation between antibody affinity to HA1 (but not HA2) and the cross-clade H5N1 neutralization titers was observed.

## Materials and Methods

### H5N1 Prime-boost Study Design

#### Ethics Statement

This open-label study was done from May to December, 2007 at the Leicester Royal Infirmary, United Kingdom. (ClinicalTrials.gov, NCT00478816). The United Kingdom Medicines and Human Products Regulatory Agency, an independent ethics committee, and University Hospitals Leicester approved the study. All samples were de-identified. The study in CBER was conducted under Research Involving Human Subjects (RIHSC) exemption 03–118B.

**Figure 1:**
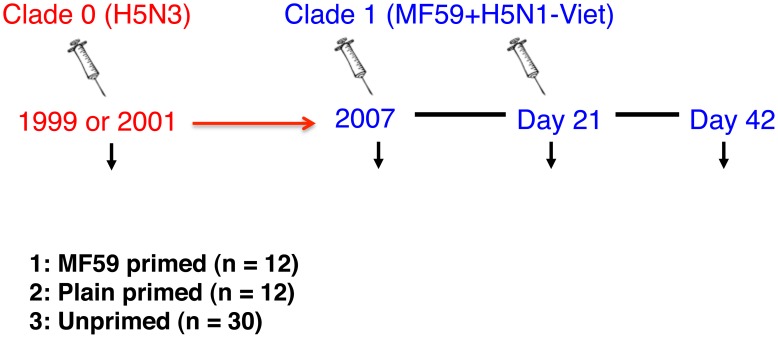
H5N1 prime-boost vaccine trial design. Schematic design of the heterologous H5N3 prime- H5N1boost vaccine immunization is shown and was previously described [1]. The number of individuals in each group: Gp1 = 12; Gp2 = 12; Gp3 = 30.

The outline of the study is shown in Fig. 1 and was also described in previous publications[1] [2]. Briefly, 7.5 µg of MF59-adjuvanted clade 1 H5N1 vaccine was administered to unprimed subjects **(N = 30)** and subjects who had been immunized in 1999 or 2001 with MF59-adjuvanted (N = 12) or non-adjuvanted (N = 12) clade 0 A/duck/Singapore/1997 (H5N3) vaccine [5] [6]. All subjects received 2 doses, 21 days apart, of 7.5 µg of H5 hemagglutinin in MF59-adjuvanted vaccine by intramuscular injection into the deltoid of the nondominant arm (days 0 and 21). MF59-adjuvanted surface-antigen vaccine containing 7.5 µg of hemagglutinin derived from H5N1-A/Vietnam/1194/2004 virus was used. MF59 is a registered trademark of Novartis AG.

### Affinity Measurements by Surface Plasmon Resonance (SPR)

Steady-state equilibrium binding of post-H5N1 human vaccine sera was monitored at 25°C using a ProteOn surface plasmon resonance biosensor (BioRad)[7]. The rHA0-His_6_, rHA1-His_6_ or rHA2-His_6_ protein sequence for the H5N1- A/Vietnam/1203/2004 influenza strain (were identical to the boosting vaccine strain; H5N1-A/Vietnam/1194/2004) was coupled to a GLC sensor chip with amine coupling with 500 resonance units (RU) in the test flow cells. Samples of 60 µl sera at 10-fold & 100-fold dilutions were injected at a flow rate of 30 µl/min (120-sec contact time) for association, and disassociation was performed over a 600 second interval (at a flow rate of 30 µl/min). Responses from the protein surface were corrected for the response from a mock surface and for responses from a separate, buffer only injection. Binding kinetics for the human vaccine sera and the data analysis were calculated with BioRad ProteOn manager software (version 2.0.1). Antibody off-rate constants, which describe the fraction of antigen-antibody complexes that decay per second, were determined directly from the serum/plasma sample interaction with rHA0, rHA1 or rHA2 protein using SPR in the disassociation phase as described before[7] and calculated using the BioRad ProteOn manager software for the heterogeneous sample model. Off-rate constants were determined from two independent SPR runs.

### Neutralization Assay

Viral-neutralizing activity was analyzed in a microneutralization (MN) assay based on the methods of the pandemic influenza reference laboratories of the Center for Disease Control and Prevention (CDC). Antibodies by MN were measured at the Centers for Disease Control and Prevention, Atlanta with wild-type A/Hong Kong/156/97, A/Vietnam/1194/2004, clade 1 variant A/Cambodia/R04050550/2007, A/Indonesia/5/2005, A/Turkey/15/2006, and A/Anhui/1/2005 (H5N1) strains and A/duck/Singapore/1997 (H5N3) in enhanced BSL3. For MN, sera were tested at an initial dilution of 1∶20, and those that were negative were assigned a titer of 1∶10. All sera were tested separately and in duplicate, and the geometric mean value was used for analysis.

### Statistical Analyses

Differences between groups were examined for statistical significance using Student’s t-test. No pretest was conducted across all groups before applying a student t test for pairwise comparisons. An unadjusted *p-value* less than 0.05 was considered to be significant. Spearman correlations are reported for the calculation of correlations between off-rate and MN titers.

## Results

### Heterologous Boost with MF59-adjuvanted H5N1 Subunit Vaccine Results in Higher HA1 Binding Antibodies in Individuals Previously Primed with MF59-adjuvanted H5N3 Vaccine Compared with Unadjuvanted H5N3 Prime or Unprimed Individuals

In previous publications we demonstrated an increase of broadly cross-reactive antibodies after boosting of individuals primed with H5N3-MF59 vaccine (A/duck/Singapore/1997, clade 0-like) during 1999 or 2001 with MF59-adjuvanted H5N1 (A/Vietnam/1194/2004, clade 1) vaccine [2] [1]. The impact of the H5N3 priming was particularly of interest since the time gap between the prime and boost was 6 or 8 years. Importantly, it was found that the heterologous prime boost resulted in significant expansion of cross reactive antibody titers that neutralized diverse clades of highly pathogenic H5N1, suggesting persistence of long term immunological memory (Table 1). The breadth of cross-neutralization was seen in individuals primed with either MF59-adjuvanted or unadjuvanted H5N3 subunit vaccine (Table 1 top vs. middle row). However, the geometric mean titers (GMTs) against all H5N1 viruses were higher in individuals that were primed with the MF59 adjuvanted H5N3 vaccine. The response rates of individuals that received only two doses of MF59-adjuvanted H5N1 (at 7.5 µg HA/ml) without H5N3 priming were low and did not reach the desired seroconversion rates for most H5N1 viruses.

**Table 1:** Geometric Mean and Standard deviations for the end-point microneutralization titers of three vaccine groups against diverse H5N1 virus strains.

	A/Duck	A/Vietnam	A/Indonesia	A/Turkey	A/Anhui	A/Cambodia	AHong Kong
	(H5N3)	(Clade 1)	(Clade 2.1)	(Clade 2.2)	(Clade 2.3.4)	(Clade 1 variant)	(Clade 0)
**MF59-Adj.**	5298.9±5386.2^a^	1051.3±976.8	894.2±867.1	2088.2±1259.1	1584.5±1760.1	427.6±338.6	4864.6±4433.9
	(100%)^b^	(100%)	(100%)	(100%)	(100%)	(100%)	(100%)
**Non-Adj.**	5070.7±5080.8	416.9±330.9	474.1±606.5	978.4±653.7	662.8±686.1	263±361.8	2301.3±1844.8
	(100%)	(100%)	(100%)	(100%)	(100%)	(100%)	(100%)
**Unprimed**	195.6±255.9	75.5±73.9	61.4±72.2	81.5±68.9	49.9±58.9	49.9±49.9	224±274.5
	(82.7%)	(41.4%)	(44.8%)	(62.1%)	(44.8%)	(37.9%)	(86.2%)

a MN Data are shown for post-H5N1 vaccination sera collected on day 21 following single H5N1 booster vaccination from the MF59-adjuvanted primed and nonadjuvanted primed or after two vaccinations in the unprimed control vaccine group.

b Percentage of responders (fraction of subjects) demonstrating MN titers of ≥1∶40 are shown in the parenthesis.

**Figure 2:**
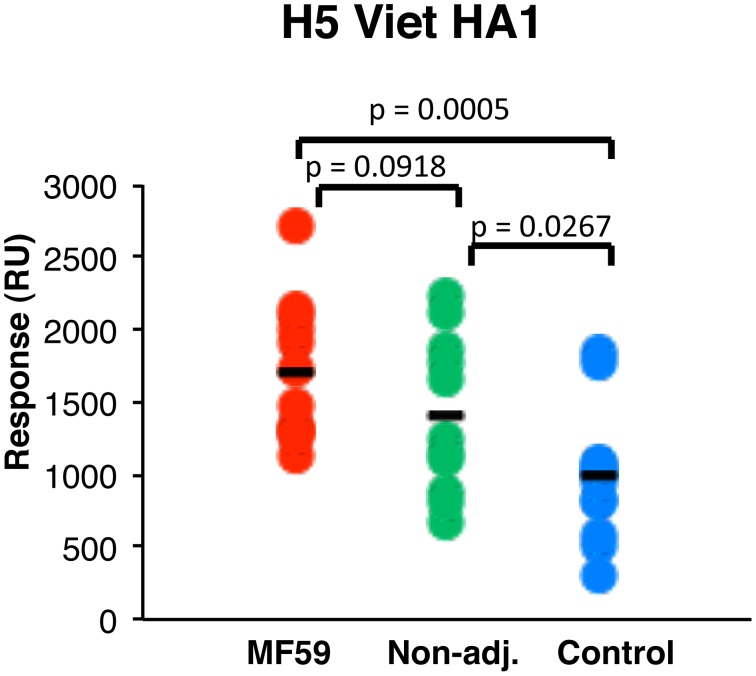
Binding of post-H5N1 vaccination polyclonal human serum to properly folded HA1 protein. Steady-state equilibrium analysis of the total binding antibodies in the polyclonal human vaccine sera to properly folded functional H5N1-A/Vietnam/1203/2004 HA1-His_6_ was measured by SPR. Ten-fold diluted individual post-boost H5N1 vaccination sera from the three vaccine groups were injected simultaneously onto HA1 immobilized on a sensor chip through the free amine group and onto a blank flow cell, free of peptide. Maximum resonance unit (Max RU) values for HA1 binding by serum antibodies obtained from multiple individuals from either MF59-adjuvanted H5N3-primed (red symbols; average-1703 RU) or unadjuvanted H5N3-primed (green symbols; average-1408 RU) on day 21 following single booster vaccination or unprimed controls after 2 doses of MF-59-adjuvanted H5N1 vaccine (blue symbols; average-982 RU). The binding antibodies in the H5N3-primed vaccine groups were significantly higher compared to the unprimed vaccine group.

The goal of the current study was to evaluate the impact of MF59 adjuvant during priming and role of heterologous prime-boost on quality of humoral immune response in the different vaccine groups using Surface Plasmon Resonance (SPR) based real time kinetics assay as previously described [3] [7]. For coating of the SPR chips we captured properly folded recombinant HA1 (amino acids 1–320; globular head) and HA2 (stalk) proteins from the boosting vaccine strain (H5N1-A/Vietnam/1203/2004) expressed in bacterial system and purified under controlled refolding conditions [3] [8]. As a control, complete intact hemagglutinin H5N1-HA0 was also used in these assays. Proper folding of recombinant proteins used in the SPR assay were confirmed by binding to conformation sensitive human MAbs generated from H5N1 infection survivors as previously described [9]. Total polyclonal sera antibody binding to HA1 (max RU) was measured for individual sera from the three prime-boost vaccine arms. Isotype analyses confirmed that the antibodies that bound the HA1 and HA2 proteins were primarily IgG confirming class switching even in the unadjuvanted vaccine group and in the group that was not primed against H5N3 (data not shown). As can be seen in Fig. 2, broad distribution of binding to H5N1 rHA1was observed in each vaccine group. However, sera from the group that received heterologous prime-boost with MF59 included in both the priming and boosting vaccines exhibited the highest levels of binding antibody (max RU) to HA1 (Fig. 2 red circles), followed by the group that received heterologous prime with unadjuvanted H5N3 vaccine (green circles) (not statistically significant). Sera from individuals that received two doses of MF59-adjuvanted H5N1 (A/Vietnam/1194/2004) without prior priming with H5N3 (blue circles) had the lowest antibody binding to the rHA1 that was statistically different from the groups that were primed with H5N3 vaccine 6 or 8 years earlier either with MF59 (*p = 0.0005*) or without MF59 (*p = 0.0267*).

### Heterologous Prime-boost Vaccination Elicits Higher Affinity Antibodies against HA1 (but not HA2) Compared with Antibodies from Unprimed Individuals

**Figure 3:**
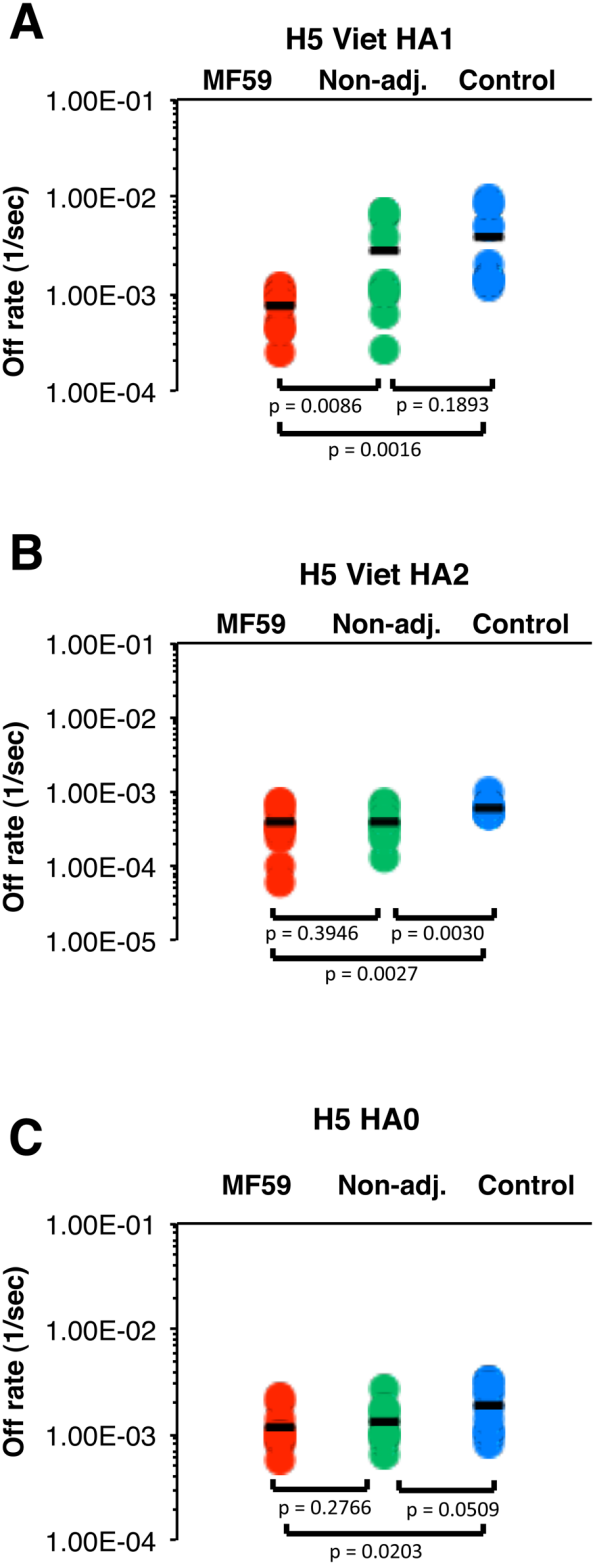
MF59-H5N3 priming enhances antibody affinity (slower off-rates) to H5N1-HA1 (but not HA2) following a single heterologous H5N1 booster vaccination. (A–C) SPR analysis of post-H5N1 vaccinated human sera from all the H5N1-A/Vietnam/1203/2004 responders (MN≥1∶40) in three vaccine groups was performed with properly folded HA1 (A), HA2 (B) domains or HA0 (C) from H5N1 A/Vietnam/1203/2004 strain. Off rates of polyclonal antibodies on day 21 following a single H5N1 booster vaccination from the MF59- adjuvanted H5N3 primed (red symbols) or unadjuvanted H5N3 primed (green symbols) individuals, or after two vaccinations with MF59-adjuvanted H5N1 in previously unprimed controls (blue symbols) are shown. Antibody off-rate constants that describe the fraction of antibody-antigen complexes decaying per second were determined directly from the serum sample interaction with rHA1 (1–320) protein, rHA2 (331–480) or rHA0 (1–513) using SPR in the dissociation phase. Serum antibody off-rate constants were determined as described in Materials and Methods. *p-values* of less than 0.05 were considered significant.

We have previously demonstrated that it is possible to measure the avidity of antibodies in the polyclonal human sera by measurements of steady state binding dissociation rates of antigen-antibody complexes using the SPR technology for affinity measurement of vaccine induced immune responses [10] [7] [8] [11], [12]. In the current study, it was important to determine if the earlier priming with H5N3 vaccine resulted in induction of long term memory B cells and the role of MF59 adjuvant during priming on antibody affinity maturation. To that end, we determined the off-rates of binding plasma antibodies from all individuals in the study, using SPR chips coated with rHA1, rHA2, or rHA0 from H5N1 A/Vietnam/1203/2004 (HA sequence similar to the boosting vaccine strain). As can be seen in Figure 3A, a significant difference in anti-HA1 polyclonal serum antibody off-rates was found between unprimed vs. remotely primed individuals (blue circles vs. green and red circles). The antibody binding off rates to H5N1-rHA1 of the polyclonal sera from heterologous MF59-H5N3 (MF59) primed followed by single MF59-H5N1 boosted vaccinees were significantly lower (∼1 log)) than those in sera from unprimed (control) MF59-H5N1 vaccinated individuals (red vs. blue circles, *p = 0.0016*). The anti-HA1 antibody off-rates were also significantly lower in individuals primed with MF59-H5N3 (MF59) compared with unadjuvanted H5N3 primed (Non-Adj.) individuals (red vs. green circles, *p = 0.0086*). In contrast, when chips were coated with rHA2 (panel B) or rHA0 (panel C), high binding affinities were measured for all three groups (dissociation off-rates close to 1×10^−3^ per sec). Statistical differences were found between the off rates of primed vs. unprimed sera, but the differences between MF59-H5N3 compared to unadjuvanted H5N3 primed groups were not as apparent as against the rHA1 domain (Panels B–C vs. A). These findings could be explained by the very high conservation between the HA2 of H5N1 and seasonal H1N1 strains leading to high affinity antibodies in most adults targeting the conserved HA2 stalk domain as described before [3]. It is clear that the avidity of binding to intact HA0 is combination of antibodies binding to various antigenic sites within HA1 and HA2 and primarily driven by higher affinity antibodies, which are mostly against the conserved HA2 domain. These findings suggest the antibody affinity maturation against different antigenic sites occur independently within the influenza HA and underscores the need to measure antibody affinity separately against the different antigenic domains within influenza hemagglutinin; HA1 globular domain, which is the most variable among types/subtypes, and the more conserved HA2 stalk domain. In all studies to date the biggest impact of adjuvants and prime-boost approaches was on the affinity maturation against the HA1 globular domain. However, in the current study, a modest increase in antibody affinity against the HA2 stalk was also observed in the heterologous prime-boost groups compared with the unprimed group (panel B).

### High Affinity anti-HA1 Antibodies after Heterologous Prime-boost Vaccination Correlate with Broadening of H5N1 Cross-clade Neutralization

**Figure 4:**
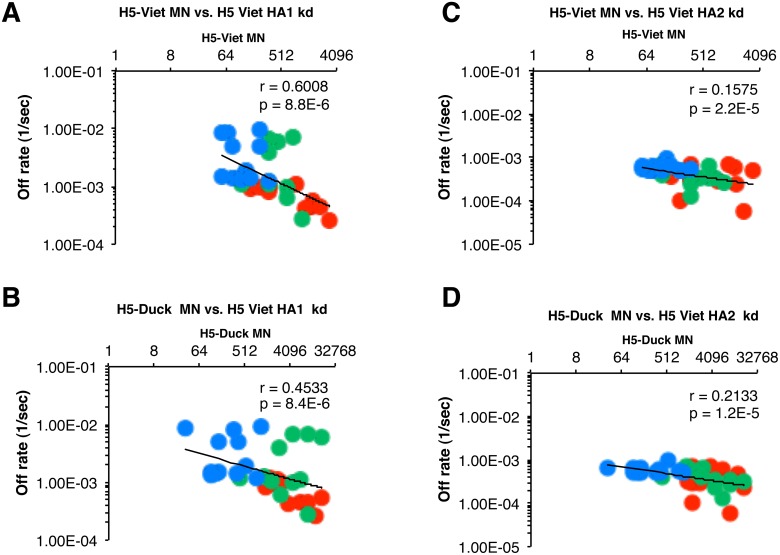
Serum antibody off-rates to H5-Viet-rHA1 (but not rHA2) following heterologous prime-boost strongly correlate with the *in-vitro* neutralizing capacity against the homologous H5 vaccine viruses. Antibody off-rate constants were determined directly from the plasma sample interaction with H5N1 rHA1 or rHA2 protein using SPR in the dissociation phase. SPR analysis of post-boost vaccination human sera from all vaccine groups included in the vaccine trial was performed with rHA1 (A and B) or rHA2 (C and D) of the H5N1- A/Vietnam/1203/2004 strain. Each symbol represents one individual. Serum samples on day 21 following single H5N1 booster vaccination from the MF59-H5N3 adjuvanted primed (red symbols) or unadjuvanted H5N3 primed (green symbols) or after two MF59-H5N1 vaccinations in the unprimed control (blue symbols) vaccine group is shown. Antibody affinity of post-H5N1 vaccinated human sera against HA1 (but not HA2) of H5N1- A/Vietnam/1203/2004 correlated with the homologous MN titers against the A/Vietnam/1203/2004 (H5N1) booster vaccine virus (A) as well as A/duck/Singapore/1997 (H5N3) priming vaccine virus (B).

**Figure 5:**
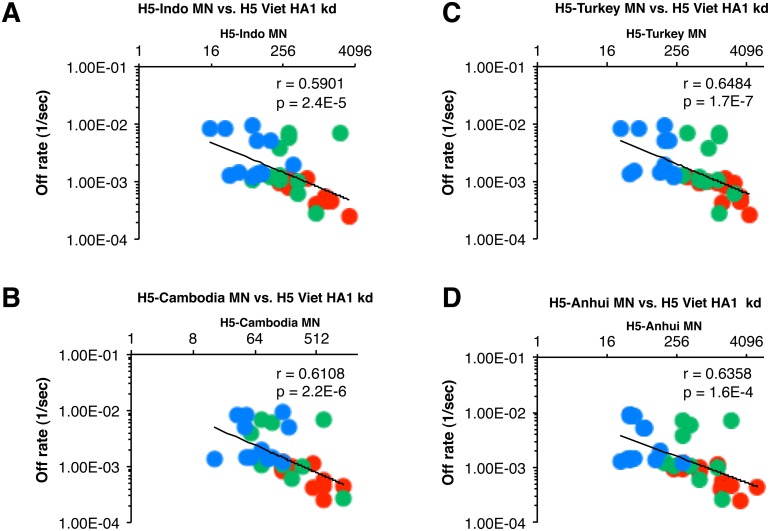
Serum antibody off-rates to H5-Viet-rHA1 (but not HA2) following prime-boost strongly correlates with the *in-vitro* cross-neutralizing capacity against diverse H5N1 strains. End-point neutralizing antibody titers of post-H5N1 vaccination sera are plotted on the X-axis. Antibody off-rate constants that describe the fraction of antibody-antigen complexes decaying per second were determined directly from the plasma sample interaction with rHA1 protein using SPR in the dissociation phase are shown on Y-axis. SPR analysis of post-boost vaccination human sera from responders (MN≥1∶40) of all vaccine groups included in the vaccine trial was performed with rHA1 of the H5N1-A/Vietnam/1203/2004 strain. Serum samples on day 21 following single MF-59-H5N1 booster vaccination from the MF59-H5N3 adjuvanted prime (red symbols) or unadjuvanted H5N3 prime (green symbols) or after two MF59-H5N1vaccinations in the unprimed control (blue symbols) vaccine group are shown. Antibody affinity of post-H5N1 vaccinated human sera against HA1 of H5N1-A/Vietnam/1203/2004 strongly correlated with the *in-vitro* heterologous cross-neutralizing MN titers against H5N1 clade 2.1- A/Indonesia/5/2005 (A), clade 1 variant- A/Cambodia/R04050550/2007 (B), clade 2.2- A/Turkey/15/2006 (C), and clade 2.3.4- A/Anhui/1/2005 (D) strains.

The biggest challenge to pandemic preparedness is to select vaccine modality and composition that is most likely to provide cross-protection against emerging H5N1 strains that may adapt for human-to human transmission. Therefore it was important to determine if the higher affinity of antibodies against H5N1-HA1 (i.e., lower off-rates) found with polyclonal sera from previously H5N3-primed individuals correlated with the multi-clade H5N1 neutralizing titers (shown in Table 1). The neutralization titers against each H5N1 virus strain were plotted against the antibody off-rates of the individual sera measured against rHA1 or rHA2 derived from H5N1-A/Vietnam/1203/2004 (Fig. 4). Very good inverse correlation was found between the HA1 (A/Vietnam) binding off-rates and the neutralization titers against the H5N3 priming vaccine strain with r = 0.4533 (Fig. 4B) as well as the booster H5N1 vaccine strain with r = 0.6008 (Fig. 4A). In contrast, the correlation between neutralization titers and HA2 (A/Vietnam) binding off rates was not obvious with r = 0.157 and r = 0.2133 (Fig. 4C, D, respectively). Among the vaccine groups, those individuals that received both priming and boosting with MF59 adjuvanted vaccine (red circles) tended to aggregate in the right-lowest part of the correlation curves indicating lowest off-rates (i.e. highest affinity) and highest neutralization titers. The plasma from individuals receiving two MF59-adjuvanted H5N1 doses with no prior priming (blue circles) tended to aggregate in the top left part of the curves. Most importantly, plasma from individuals in the heterologous prime-boost vaccination groups demonstrated broad cross-neutralization of H5N1 clades/strains not included in either the prime or the boost vaccine (Table 1). Some of the cross-clade neutralization titers were even higher than the homologous titers, suggesting the expansion/maturation of heterosubtypic long-term B memory cells. As can be seen in Fig. 5 strong inverse correlation was found between the anti-HA1 antibody off-rates against the H5N1 vaccine strains and the cross-neutralization titers of H5N1 Indonesia (clade 2.1) (panel A), H5N1 A/Cambodia (clade 0) (panel B), H5N1 A/Turkey (clade 2.2) (panel C), and H5N1 A/Anhui (clade 2.3) (panel D) for the post-vaccination sera from the individuals in all the vaccine groups. In the case of heterologous prime-boost, 100% plasma reached titers of ≥1∶40 against the heterosubtypic H5N1 strains (Table 1 top and middle row), while the unprimed group that received two doses of MF59 adjuvanted H5N1 (A/Vietnam) vaccine did not reach 100% seroprotection rates against any of the H5N1 strains (Table 1). The GMT against the heterosubtypic H5N1 strains tended to be higher in vaccinees that received both priming and boosting with MF59-adjuvanted vaccines and their post-vaccination serum antibodies demonstrated the highest anti-HA1 antibody affinities.

Together these data provided important information on the quality of the antibodies generated after heterologous boosting and role of MF59 adjuvant on antibody affinity maturation in the priming regimen. The strength and breadth of virus neutralization correlated with the levels and affinity of antibodies binding to the HA1 domain of the boosting H5N1 vaccine strain.

## Discussion

The current study demonstrated that the improved recall response against H5N1 vaccine virus strain (A/Vietnam, clade 1) in individuals exposed to prior vaccination with heterologous strain (H5N3) (A/Duck) 6 or 8 years earlier strongly correlated with post-vaccination serum antibody binding to recombinant HA1 from the boosting H5N1 virus strain. Furthermore, antibody off-rates for HA1 but not HA2 were strong predictors of cross clade H5N1 virus neutralization titers. These data extend previous reports from the same prime-boost study[2] [1] and may shed light on the importance of adjuvant in generating long term memory B cells, promoting antibody affinity maturation with expanded recognition of cross-clade H5N1 virus strains.

Long lived maintenance of humoral immunity is provided by a combination of long lived antibody plasma cells (LLPC) and memory B cells [13] [14] [15]. Multiple studies were conducted to better understand the differences between long living plasma cells and memory B cells (reviewed by Tarlinton and Good-Jacobson [16]). In a mouse model of West Nile Virus (WNV) infection it was demonstrated that LLPC generated during the primary response against WNV Domain III of the E glycoprotein were uniformly specific for a dominant neutralizing determinant of the virus, with poor ability to block infection of variant viruses. On the other hand, the memory B cell compartment contained cells with reactivity to viral variants, and after re-stimulation produced some antibodies with higher affinity for the variant virus compared with the inducing viral antigen (*heteroclitic antibodies*). The authors concluded that the memory B cell compartment included B cells with specificities not found in the LLPC compartment. Furthermore, the expanded specificities were associated with germinal center (GC)-derived isotype switched memory B cells [17]. Similar scenario is likely to take place during vaccination or exposure to influenza [18]. Therefore, prime-boost vaccination approaches that drive the GC expansion of memory B cells with somatic hyper mutations (SHM) of the BCR H/L chains will result in both affinity maturation and repertoire expansion that includes heterosubtypic specificities as demonstrated in the current and previous studies. Importantly we have demonstrated a direct correlation between cross-clade neutralization and the development of high affinity antibodies during human clinical trials with either adjuvanted vaccines or DNA-prime MIV boost vaccination strategies [3] [12].

The group of individuals that were primed with unadjuvanted H5N3 presented a more heterogeneous profile in terms of antibody off rates and neutralization titers. Indeed, in some individuals, high neutralization titers were observed with high titers of lower affinity antibodies. This may be due to the very long time between the priming and heterologous boost. In some individuals antibody diversification may occur without affinity maturation either inside or outside GC and may be impacted by host factors that require further studies. However, the presence of adjuvant during the initial vaccination resulted in more uniform antibody affinity maturation after subsequent heterologous boost.

While most of the affinity maturation was observed in antibodies targeting the HA1 globular head, an increase in binding avidity (slower off-rates) was also found against the HA2 stalk domain. We cannot exclude the possibility that broadly neutralizing antibodies targeting the stalk region may contribute to the expanded cross-protection *in vivo* through post receptor-binding events including fusion inhibition or Fc-mediated antibody-dependent cell cytotoxicity (ADCC) resulting in killing of infected cells, as recently reported [19], [20]. However, these types of antibodies are not commonly measured in the MDCK (cells lacking Fc receptor) based microneutralization assay used in the current study.

Oil in water adjuvants including MF59 (Novartis Vaccines and Diagnostics) and AS03 (GSK) were shown to significantly improve the humoral immune responses against avian influenza and also to induce broad cross-clade neutralizing activities against diverse clades of H5N1 [21] [4] [22] [23]. Furthermore, it was demonstrated that an increase in the number of influenza-specific CD4^+^ T cells after a single dose of MF59-adjuvanted H5N1 vaccine correlated with the rise and long term maintenance of protective antibody titers against avian H5N1 influenza that reached protective titers only after the second vaccination [24]. More recently it was demonstrated that T_FH_-like cells with partial phenotype of GC T_FH_ (ICOS^+^, PD1^+^, IL-21^+^) can be found in the blood of humans after vaccination with MF59-adjuvanted influenza vaccine. These cells can exert helper function to influenza-specific B cells in CD40L- and IL-21- dependent fashion [25]. Their frequencies correlated with the influenza-specific B cell responses. The added value of the adjuvant during the heterologous boost was not addressed in the current study (no unadjuvanted H5N1 A/Vietnam arm), but should be addressed in subsequent studies.

In conclusion, the current study provides explanation for the increased antibody titers and the superior H5N1 cross neutralization responses against variant highly pathogenic avian influenza strains in vaccinated individuals that were previously primed with heterologous avian influenza vaccine, especially if combined with the MF59 adjuvant. It is likely that the adjuvant increase GC formation, increase the frequency of influenza specific T_FH_ that can re-enter old GC and possibly re-distribute between the blood and GCs after each vaccination. These elevated numbers of influenza-specific T_FH_ subsets in terms are likely to drive the expansion of memory B cells (rather than LLPC) resulting in a more robust long term memory pool of memory B cells with high affinity BCRs that can recognize variant influenza strains not included in the original vaccination. In the case of exposure to novel avian influenza strains, a robust recall response of such memory B cells can be the first line of defense against a pandemic influenza threat with diverse H5N1 strains.
